# The Three-Dimensional Distribution of αA-Crystalline in Rat Lenses and Its Possible Relation to Transparency

**DOI:** 10.1371/journal.pone.0023753

**Published:** 2011-08-31

**Authors:** Guido A. Zampighi, Lorenzo Zampighi, Salvatore Lanzavecchia

**Affiliations:** 1 Department of Neurobiology, David Geffen School of Medicine at UCLA, Los Angeles, California, United States of America; 2 Department of Physiology, David Geffen School of Medicine at UCLA, Los Angeles, California, United States of America; 3 Jules Stein Eye Research Institute, David Geffen School of Medicine at UCLA, Los Angeles, California, United States of America; 4 Dipartimento di Chimica Strutturale, Univesità degli Studi di Milano, Milan, Italy; Dalhousie University, Canada

## Abstract

Lens transparency depends on the accumulation of massive quantities (600–800 mg/ml) of twelve primary crystallines and two truncated crystallines in highly elongated “fiber” cells. Despite numerous studies, major unanswered questions are how this heterogeneous group of proteins becomes organized to bestow the lens with its unique optical properties and how it changes during cataract formation. Using novel methods based on conical tomography and labeling with antibody/gold conjugates, we have profiled the 3D-distribution of the αA-crystalline in rat lenses at ∼2 nm resolutions and three-dimensions. Analysis of tomograms calculated from lenses labeled with anti-αA-crystalline and gold particles (∼3 nm and ∼7 nm diameter) revealed geometric patterns shaped as lines, isosceles triangles and polyhedrons. A Gaussian distribution centered at ∼7.5 nm fitted the distances between the ∼3 nm diameter gold conjugates. A Gaussian distribution centered at ∼14 nm fitted the Euclidian distances between the smaller and the larger gold particles and another Gaussian at 21–24 nm the distances between the larger particles. Independent of their diameters, tethers of 14–17 nm in length connected files of gold particles to thin filaments or clusters to ∼15 nm diameter “beads.” We used the information gathered from tomograms of labeled lenses to determine the distribution of the αA-crystalline in unlabeled lenses. We found that αA-crystalline monomers spaced ∼7 nm or αA-crystalline dimers spaced ∼15 nm center-to-center apart decorated thin filaments of the lens cytoskeleton. It thus seems likely that lost or gain of long-range order determines the 3D-structure of the fiber cell and possible also cataract formation.

## Introduction

To attain transparency, the lens underwent a series of evolutionary adaptations that include the elimination of blood vessels from its interior and the accumulation of massive quantities (600–800 mg/ml) of a heterogeneous group of small molecular weight (20–30 kDa) proteins, called crystallines, in the cytoplasm of highly elongated fiber cells [1]–[4]. Human lenses express twelve primary crystalline gene products and two truncated forms [5]–[8]. A major unanswered question is how these fourteen soluble proteins are organized to bestow the lens with its unique optical properties and the changes induced by cataracts, the principal cause of blindness worldwide.

A large body of experimental evidence suggests that crystallines form multi-subunit assemblies that are organized with “short-range” order of dense solutions in the cytoplasm of fiber cells [9]–[12]. Evidence suggesting this organization includes: a) the “amorphous” structure of the cytoplasm of fiber cells observed in conventional electron microscopy studies [13]–[16], and b) the absence of long-range order observed in solutions of purified crystallines [17]–[19]. Crystallines organized as dense solutions predict that cataracts involve non-specific protein aggregation and the formation of light-scattering particles. Yet, studies of fractions isolated from chick and later mammalian lenses reveal a unique type of protein assembly, called the “beaded” filament, which is difficult to reconcile with the short-range order of dense solutions.

Structurally, “beaded” filaments contain cores decorated with particles (“beads”) spaced 21–24 nm center-to-center apart [20]–[22]. Most investigators agree that proteins of the “intermediate” filament (IF) family, called cytoskeletal protein 49, (CP49 or “phakinin”), and cytoskeletal protein 115 (CP115 or “filensin”) comprise the core of the “beaded” filament [23]. A current molecular model depicts “beaded” filaments comprised of four phakinin protofilaments surrounded by filensin/phakinin shells. In this model, the C-terminal domain of filensin represents the “bead” that repeats alongside the axial direction [24]. A competing model proposes that the “bead” is an assembly comprised of multiple subunits of the αA-crystalline evenly spaced along the filensin/phakinin core [18], [19]. Independent of whether the “bead” represents the C-terminal domain of filensin or a multi-subunit assembly of the αA-crystalline, the presence of an ordered structure raises the possibility that lost or gain of long-range order determines the 3D-structure of the fiber cell and possible also cataract formation.

Unanswered questions in the lens structure and function are the protein composition of the repeating “beads” and how their 3D-organization can be reconciled with the “amorphous” structure of the cytoplasm of the fiber cell. To answer these questions, we have reconstructed rat lenses labeled with anti-αA-crystalline conjugated to gold particles (∼3 nm and ∼7 nm diameter) and from unlabeled lenses. We hypothesized that if “beads” are multi-subunit assemblies of the αA-crystalline, the smaller gold particles would form clusters centered on the ∼15 nm in diameter particles but the larger gold particles would be arranged in lines or rows spaced 21–24 nm center-to-center apart.

Our study strongly supports the hypothesis that in rat lens fiber cells the αA-crystalline decorates the filensin/phakinin filamentous core as monomers spaced ∼7 nm apart or as dimers spaced ∼15 nm apart (the “αA-crystalline motif”). These motifs form highly ordered 3D-matrices that enfold the massive quantities of crystallines expressed in fiber cells. It thus seems likely that lens transparency and perhaps also cataract formation depend on unanticipated high degrees of long-range order in the lens cortex and nucleus.

## Results

### The “projected” structure of fiber cells

**Figure 1:**
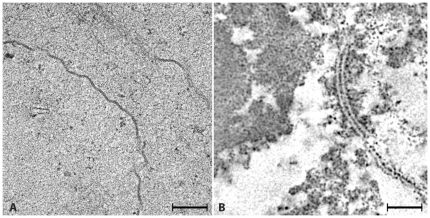
Projected Structure. **Panel A** shows a low magnification view of three cortical fiber cells from the equatorial region of a rat lens. The plasma membranes appear as electron-dense bands. In contrast, the cytoplasm appears unstructured with occasional clusters of electron-dense particles. At this magnification, the cytoskeleton assemblies referred as “beaded” filaments are not present. **Panel B** shows a higher magnification view of a region of plasma membrane surface to demonstrate the characteristic pentalamellar structure of gap junctions. Bar: A = 0.2 µm, B = 40 nm.

Our studies focused on “developed” fibers; a group of highly elongated cells that lack most cytoplasmic organelles, including nuclei [3]. At low magnification, the developed fibers contain an “amorphous” cytoplasm limited by distinct electron dense bands at the surface (Fig. 1A). At higher magnification, the bands appeared as pentalamellar structures 12–15 nm in thickness that at regions split into ∼5 nm in thickness unit membranes (Fig. 1B). These pentalamellar structures represent regions where the plasma membranes of neighboring fiber cells form the “gap junctions,” the organelles that function in lens cell-to-cell communication [25], [26]. In contrast, the cytoplasm of these developed fibers appeared “amorphous” or unstructured with no indication of the ordered “beaded” filaments identified in fractions isolated from lens tissues [20], [22].

### The Method

**Figure 2:**
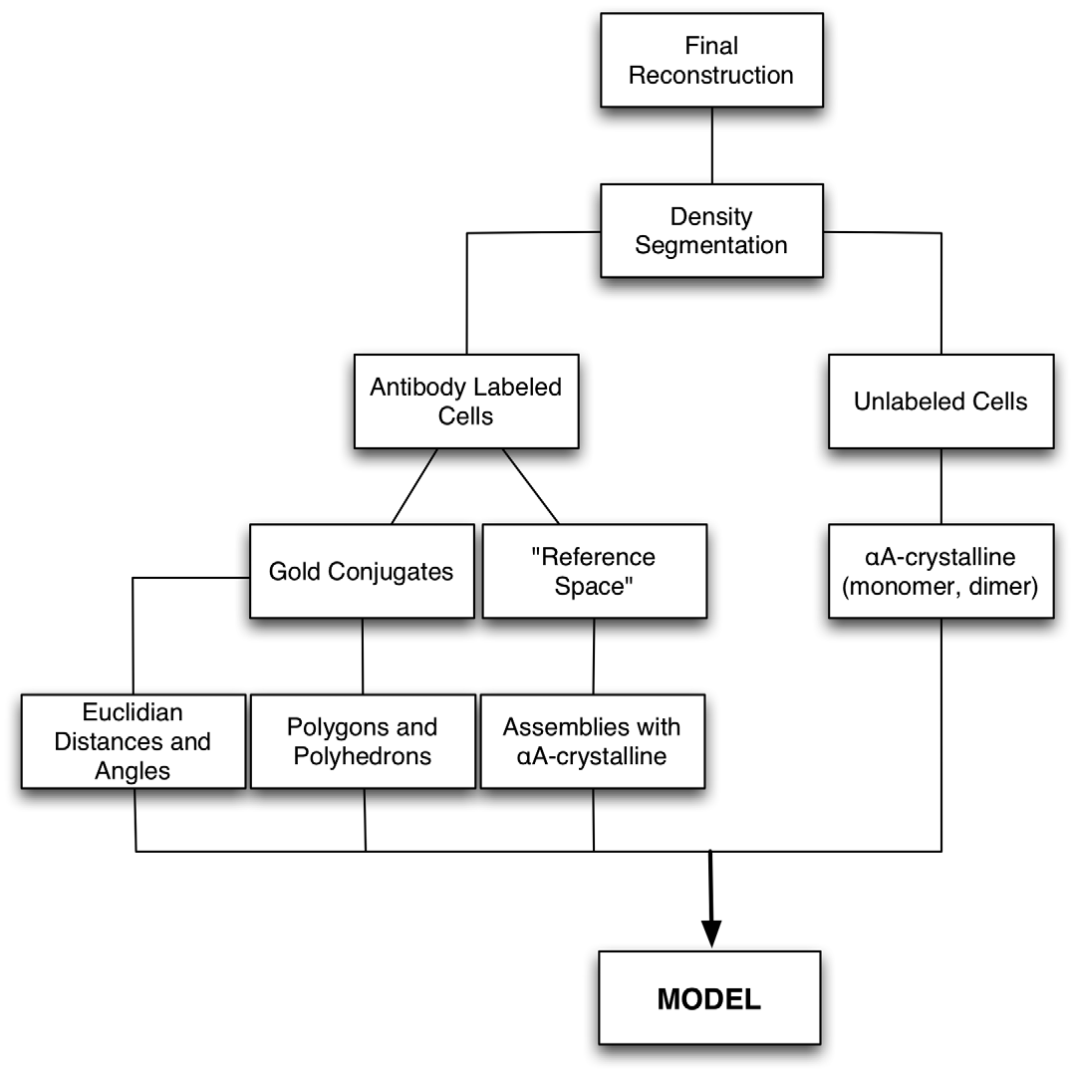
Flow Chart of the Experimental Protocol.

The flow chart (Fig. 2) of the experimental protocol recapitulates the method used to determine the 3D-distribution of the αA-crystalline in fiber cells of rat lenses. In essence, the method compares tomograms calculated from fiber cells labeled with anti-αA-crystalline and from unlabeled cells. To visualize the anti-αA-crystalline complex, we used secondary antibodies conjugated to gold particles seemingly measuring ∼2 nm and ∼5 nm diameter. The smaller gold conjugates mirrored the distances and the geometric patterns of the αA-crystalline subunits within the “bead” assemblies. In contrast, the larger gold conjugates reflected the distances and geometric patterns of repeating “beads” in different regions of the cytoplasm. Since the measurements were performed in conical tomograms, the distances are Euclidian and hence they are not limited by projection artifact. The information gathered from the tomograms calculated from labeled cells was used for determining the 3D-distribution and dimensions of αA-crystalline subunits in the cytoplasm of unlabeled fiber cells.

### The 3D-structure of fiber cells labeled with anti-αA-crystalline/gold particle conjugates

**Figure 3:**
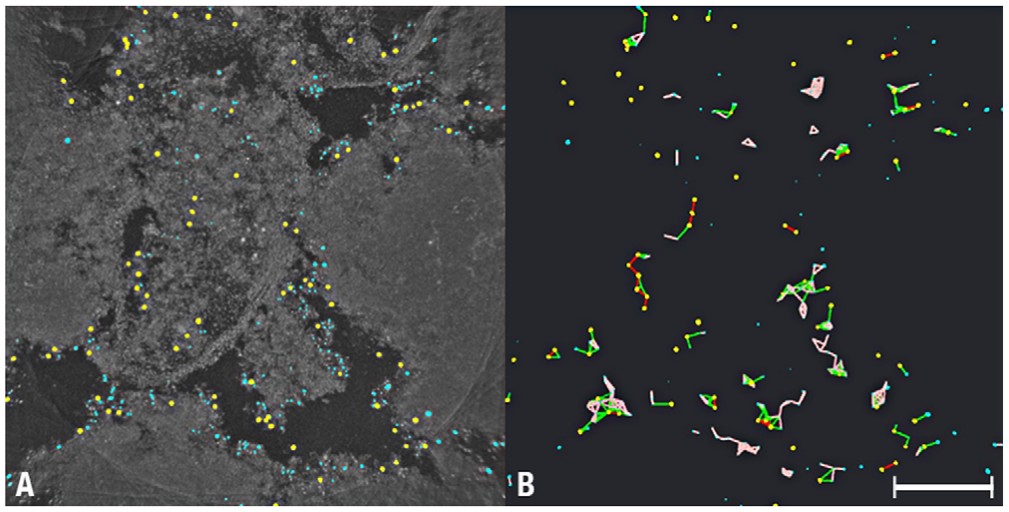
Distribution and Classification of Gold Conjugates. **Panel A** is a Z-projection of a tomogram computed at maximum intensity and presented in reverse contrast to highlight the distribution of gold conjugates in the tomogram. Depending on their diameters and peak intensities, the conjugates were classified as yellow (>4 nm diameter OR peak intensity >200) or blue (>2 nm in diameter OR peak intensity >150). **Panel B** shows the gold conjugates contained in the volume. The color-coded lines represent the Euclidean distances connecting yellow (red lines), yellow-to-blue (green lines) and blue conjugates (brown). Bar A–B: 0.1 µm.

**Figure 4:**
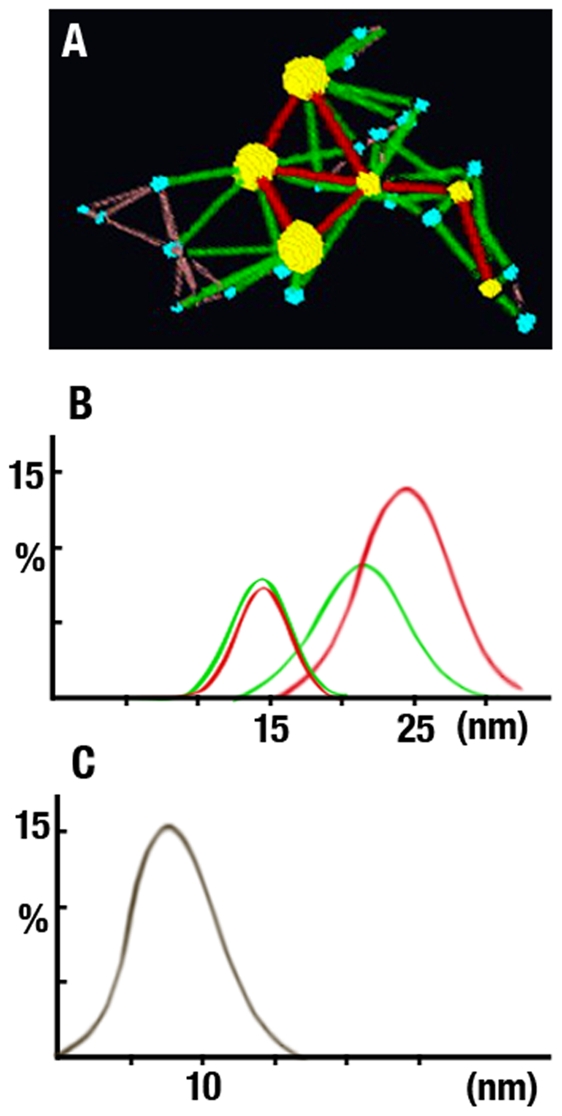
Measurement of the Euclidean Distances between Conjugates. **Panel A** shows a small region of a tomogram with six yellow (∼7 nm diameter) and nineteen blue (∼3 nm diameter) conjugates. The color-coded lines represent the Euclidian distances between yellow (red), yellow-to-blue (green) and blue (brown) conjugates. Note that independent of their diameters, the conjugates occupy the vertices of isosceles triangles. **Panels B&C** show histograms of the Euclidian distances between 107 yellow and 225 blue conjugates. **Panel B** shows that two Gaussian distributions centered at ∼14 nm and 21–24 nm fit the Euclidian distances between yellow (red) and yellow-to-blue conjugates (green). **Panel C** shows that the single Gaussian distribution centered at ∼7.5 nm fits the Euclidian distances between blue conjugates. In B and C, the x-axis plots distance in nm and the y-axis percentage.

First, we took advantage of the large amplitude contrast of gold to split the tomograms calculated from labeled cells into 3D-maps with only gold particles and 3D-maps with the electron-densities representing the allegedly “amorphous” cytoplasm of the fiber cell (the “reference space”). In the maps with the gold particles, we measured the volume of each particle, the peak intensity (8-bits intensity range 0–255) and the X,Y,Z coordinates of the mass centers (Table 1; Fig. 3A–B). The diameter of the gold particles (calculated from the volume) extended from <2 nm to >7 nm, instead of the ∼2 nm and ∼5 nm diameter classes promised by the vendor. To compute the Euclidian distances, it was thus necessary to classify the gold particles into “yellow” (diameters >4 nm OR peak intensity >200; n = 107) and “blue” (diameter >2 nm OR peak intensity >150; n = 225) classes (Fig. 4A–C; Table 1). (OR indicates the Boolean operator used for classification.) We then computed automatically the distances between the mass centers of the gold particles of the yellow, the yellow-to-blue and the blue classes.

**Table 1:** Labeled Cells.

Gold Conjugates			Euclidian Distances			
Class	Number	*Diameter (nm)	Type	Upper Limit (nm)	#Gaussian Center (nm)	Connection Number
Blue	225	3.0±0.6	Blue-Blue	10	7.5±3.5	90
			Blue-Yellow	28	13.5±2 21.0±3.5	169
Yellow	107	7±1.5	Yellow-Yellow	32	14.0±2 24.0±3.5	45

*Mean ± SD.

# Gaussian Center ± HWHM (Half Width at Half Maximum).

The Euclidian distances between the yellow-to-blue and the yellow-to-yellow gold particles were fitted by two Gaussian distributions centered at 13.5–14 nm (Half-Width at Half-Maximum; HWHM = 2.0 nm) and 21–24 nm (HWHM = 3.5 nm) (red and green curves, Fig. 4B). In contrast, a single Gaussian distribution centered at 7.5 nm (HWHM = 3.5 nm) fitted the Euclidian distances between the blue-to-blue gold particles (Fig. 4C). To visualize the geometric patterns between neighboring gold particles, we connected the mass centers with color-coded lines (Fig. 3B and 4A). This computational step revealed that the yellow conjugates formed lines or rows and the blue conjugates occupied the vertices of equilateral triangles, squares, pyramids and tetrahedrons (Fig. 4A).

**Figure 5:**
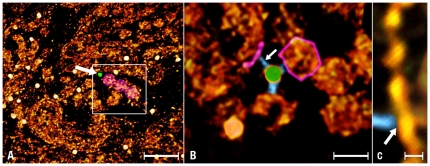
Tethers. **Panel A** is a low magnification view of a fiber cell labeled with anti-αA-crystalline/gold complexes (white discs). At this magnification, the larger conjugates (∼7 nm diameter) appear randomly scattered in the volume and the tethers that connect them to protein assemblies are not visible. To identify these tethers, small volumes (square, 128 128 47 pixels) around a central gold conjugate were cut and segmented using the watershed transform (see Methods). **Panel B** is a view of the square in A. It was rotated to find out the direction that visualized these tethers. Three tethers (blue) connected the gold conjugate (green) to protein assemblies shaped as thin filaments and spherical particles (red). **Panel C** is a higher magnification view of the thin filament colored red in B. The arrow indicates place where the tether (blue) joins the thin filament. Bar: A = 70 nm; B = 12 nm; C = 5 nm.

**Figure 6:**
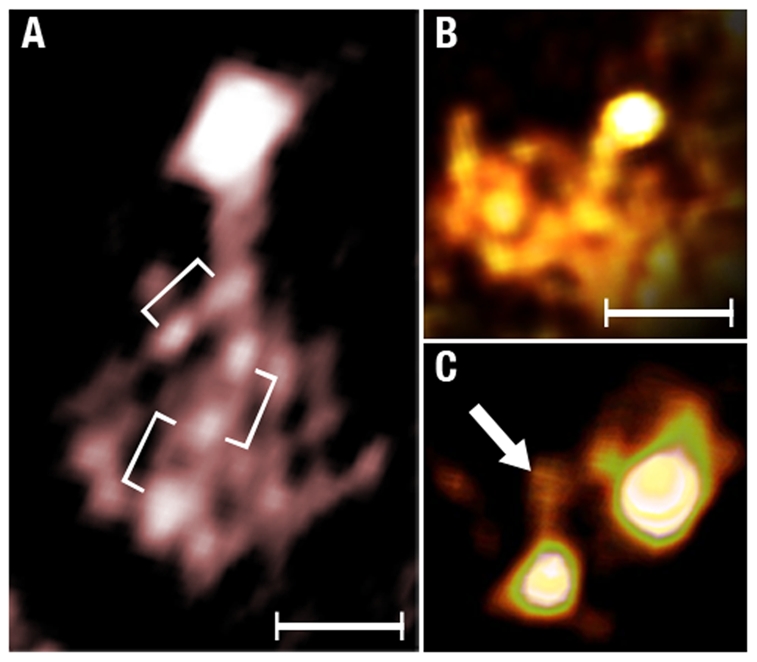
Thin Filament and “Bead” Assemblies. **Panel A** shows a thin filament decorated with three small gold conjugates (brackets) and a larger gold conjugate labels a single spherical particle (the “bead”). The inset shows a “bead” assembly labeled with four small conjugates at the vertices of a square. **Panel B** shows a single-pixels slice of a “bead” assembly connected to a large gold conjugate. The brackets indicate evenly spaced particles in the “bead.” **Panel C** shows a view of a rendered volume showing a single small gold conjugate tethered to a “bead” assembly. **Panel D** shows a large and a small gold conjugate tethered to a single “bead” assembly. The arrow points to the region where a tether joins the “bead.” To visualize these tethers, the image was computed at high intensity. Bar: A–C = 10 nm.

Second, we identified the tethers formed by the association of the primary and the secondary antibodies and followed their paths to the assemblies containing the αA-crystalline (arrows Figs. 5B–C & 6C–D). Independent of the gold particle's diameter, the tethers measured 14±3 nm in length (mean ±SD, n = 17) and terminated on assemblies shaped as thin filaments or ∼15 nm diameter “beads.” When labeling the thin filaments, the blue gold particles were spaced ∼7.5 nm center-to-center apart and the larger particles classified as yellow were spaced at double or triple this basic spacing (∼14 nm or 21–24 nm; Fig. 3B, brackets, Fig. 6A). When labeling the “beads,” the smaller blue gold particles formed clusters shaped as squares, triangles or polyhedrons (inset, Fig. 6A). In both type of assemblies, the tethers attached to small densities that were associated with the thin filaments or located in the interior of the “beads”(arrow, Fig. 5C, brackets Fig. 6B). This observation raised the possibility that the αA-crystalline was a small electron density particle bound to the cytoskeleton of the fiber cell.

### The 3D-structure of unlabeled fiber cells

**Figure 7:**
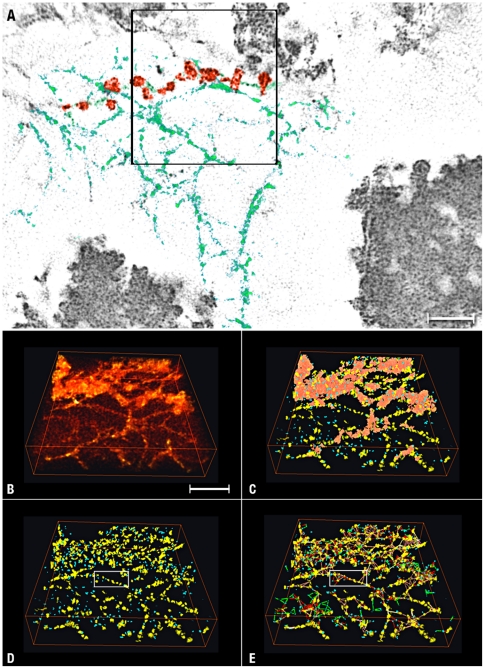
Analysis of Unlabeled Cells. **Panel A** is a single-pixel slice showing matrices comprised of filaments (green) and ∼15 nm diameter particles, referred as “bead” assemblies (red). The region inside the square (256 256 47 pixels) was segmented to reveal the repeating particles that decorate the filaments and comprise the “beads.” Bars A = 45 nm. **Panels B–E** show selected steps of the analysis. **Panel B** shows the volume in the square before segmentation. **Panel C** shows small (blue) and large (yellow) protein particles generated by segmentation. The irregular regions colored brown represent “aggregates comprised of particles >7 nm diameter. **Panel D** shows the same volume after removing the “aggregates.” **Panel E** shows the map after measuring the Euclidian distances between blue and yellow protein particles. Lines color-coded according to the dimensions of the particles connected the centers of mass of blue-to-blue, yellow-to-blue and yellow-to-yellow particles (see Table 2). Bar: 60 nm.

To find out whether the αA-crystalline comprised the small densities associated with the thin filament and the “bead” assembly, we analyzed tomograms calculated from unlabeled fiber cells. We expected that these small densities would decorate the thin filaments spaced with the same Euclidian distances and forming the same geometric patterns present in the tomograms calculated from labeled cells (Table 1). For the analysis, we calculated single-pixel slices (∼0.8 nm thickness) of entire tomograms and located smaller regions containing the thin filaments and the “beads” (square, Fig. 7A). We segmented these assemblies into a large number of densities exhibiting variable dimensions and shapes (n = 1,183, Table 2). To identify those comprised of the αA-crystalline, we classified these densities in “blue” (diameters >2 nm but <3.5 nm) and yellow (diameters >3.5 nm but <7 nm) classes. For each density, we measured the volume and determine the X,Y,Z coordinates of the mass centres (Table 2). The densities with diameters >7 nm (n = 265) were lumped in a separate class called “aggregates” (brown, Fig. 7B–E).

**Table 2:** Unlabeled Cells.

αA-crystalline Particles				Euclidean Distances			
Class	Number	*Volume (nm^3^)	*Diameter (nm)	Type	Upper Limit (nm)	#Gaussian Center (nm)	Connection Number
Blue	339	22±10	3.0±0.4	Blue-Blue	10	7.0±2.0	248
				Blue-Yellow	24	15.5±5.0	935
Yellow	116	78±20	4.0±0.3	Yellow-Yellow	32	24.0±6	265

*Mean ± SD.

# Gaussian Center ± HWHM (Half Width at Half Maximum).

From the volumes (Table 2), we estimated the molecular masses of ∼17 kDa for the blue and ∼67 kDa for the yellow density using a protein density value of 0.0013 nm^3^/Dalton [27]. From the X,Y,Z coordinates, we measured the Euclidian distances between the mass centers of the blue-to-blue, the blue-to-yellow and the yellow-to-yellow densities (Fig. 7D–E; Table 2). We found that a single Gaussian distribution centered at 7 nm (HWHM = 2; n = 248) fitted the Euclidian distances between the densities classified as blue. In contrast, two Gaussian distributions centered at 15.5 nm (HWHM = 5.0; n = 935) and 24 nm (HWHM = 6; n = 265) fitted the Euclidian distances between yellow-to-blue and yellow-to-yellow densities (Table 2). These data raised the possibility that the small evenly spaced densities labeled by the antibody/gold conjugate likely represented αA-crystalline monomers or dimers bound to the thin filaments of the cytoskeleton of fiber cells.

**Figure 8:**
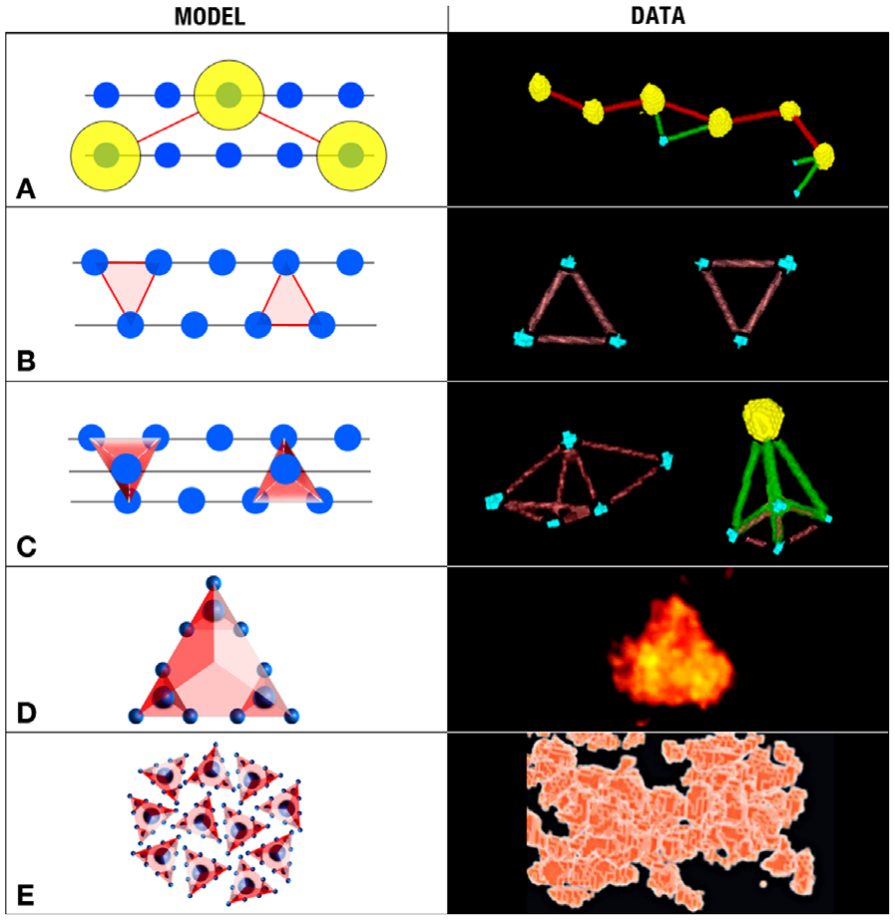
Model. **Panel A** underscores the fact that the dimers of the αA-crystalline particles (yellow) skip a position in the filament and become spaced at twice the distance (∼14 nm instead of ∼7 nm). At the right side, a kinked file of “real” yellow conjugates reflects this condition. **Panel B** shows two neighboring filaments decorated with monomers of αA-crystalline. In addition of being spaced at ∼7 nm apart, the αA-crystalline monomers occupy the vertices of isosceles triangles. At the right side, distributions of “real” blue conjugates reflect this condition. **Panel C** shows how the association of three filaments decorated with monomers at the minimum distance form tetrahedrons or pyramids. At the right side, blue and the yellow conjugates reflect this condition. **Panel D** shows a larger tetrahedron (the “bead”) formed by the assembly of smaller αA-crystalline particles. At the right side, a “real” particle reflects this condition. **Panel E** shows a region where these “beads” associate to form the larger “aggregates.” The right side panel shows a view of this type of “aggregate” where the “beads” adopt cobblestone patterns in the interior.

Finally, we studied the larger densities lumped in the class called “aggregates” (brown, Fig. 7C). Since the only constraint used in the classification was for the diameter of these densities to measure >7 nm, we expected random arrays of densities comprising the “aggregates.” To our surprise, they were arranged in cobblestone-like patterns associate along straight edges (Fig. 8E). This ordered distribution suggested that long-range order might also extend to other soluble proteins in the cytoplasm of lens fiber cells.

## Discussion

Our study provides evidence that monomers and dimers of the αA-crystalline are evenly spaced along thin filaments of the cytoskeleton in fiber cells of rat lenses (the “αA-crystalline motif”). Evidence supporting long-range order includes measurement of the Euclidian distances and the geometric patterns adopted by the αA-crystalline in tomograms calculated from labeled and unlabeled fiber cells (Tables 1&2). For example, the single line pattern reflects the monomers or the dimers of the αA-crystalline repeating alongside intermediate filaments. The isosceles triangles and polyhedrons (tetrahedrons and pyramids) patterns reflect the distribution of the αA-crystalline at regions where the intermediate filaments intersect to form 3D-matrices in the cytoplasm of these cells. It thus seems likely that the “bead” assembly is a cluster of monomers or dimers of the αA-crystalline bound to intermediate comprised of filaments filensin/phakining proteins instead of the C-terminal domains of the filensin protein hypothesized in previous models [24], [28].

To highlight the structures adopted by the cytoskeleton, we constructed models where the motifs self-assemble constrained only by the diameter of the filament and the spacing of the αA-crystalline monomer or dimer (Fig. 8, left panels). The patterns formed by the association of two motifs depend on whether the αA-crystalline associates with each filament as monomer or dimer (Fig. 8A–B). Monomers are spaced ∼7 nm center-to-center apart and often occupy the vertices of isosceles triangles or squares. In contrast, the dimers of the αA-crystalline skip one or two sites thus doubling or tripling the basic repeat period (Fig. 8A–B). Since the αA-crystalline determines the position of the “bead,” we propose that the dimers would assemble “expanded” matrices in the cortex and monomers the more “compacted” matrices in the lens nucleus.

Adding a third motif transforms the squares into pyramids and the equilateral triangles into tetrahedrons (Fig. 8C). A fourth αA-crystalline motif transforms pyramids into octahedrons (not shown) but leaves tetrahedrons unchanged because all four vertices are already occupied with αA-crystalline monomers (Figs. 7A & 8D). Further association of these polyhedrons form larger “aggregates” that despite of exhibiting substantial long-range order in three-dimensions appear as “amorphous” regions in projection (Fig. 1A). It thus seems likely that a filensin/phakinin filament decorated with monomers or dimers of the αA-crystalline is a key scaffold determining the 3D-structure of the cytoplasm of fiber cells.

The possibility of long-range order in the lens fiber cells has been foreshadowed in mass spectrometry studies of newborn human lenses [8]. These studies revealed an integral relationship between the normalized molar percentages of the 14 crystallines expressed in human lenses. The simplest explanation of this striking relationship is that crystallines exhibit a precise three-dimensional organization in the cytoplasm of fiber cells [8]. While our study strongly supports this conclusion for the αA-crystalline, additional studies will be required to characterize the polyhedral organization of the remaining crystallines in the lens.

Finally, via the association of the filensin protein with the water channel aquaporin-0 [29], the αA-crystalline motif might participate also in linking the cytoskeleton to the fiber cell plasma membrane. The motif could perform this linkage directly via filensin or other still unidentified proteins. In either case, the attachment sites are pre-determined by the Euclidian distances separating the αA-crystalline dimers (∼14 nm or ∼21 nm) in the cortex or the αA-crystalline monomers (∼7 nm) in the compact matrices of the lens nucleus. In either situation, the spacing of the αA-crystalline in the filamentous core insures that the cytoskeleton associates with the plasma membrane alongside the entire length of the fiber cell in the lens cortex and nucleus.

It thus seems likely that to attain transparency the fiber cells assemble complex three-dimensional matrices exhibiting an unexpected high degree of long-range order. The matrices subdivide the cytoplasm into small cavities that accommodate the soluble crystallines that provide the lens with its unique optical properties. In Nature, this simple yet elegant solution occurs on many occasions. For example, bees use it when constructing their hives.

Finally, using a novel method based on computational and statistical analyses of conical tomograms, we have profiled the αA-crystalline, a lens-specific chaperone, in fiber cells at ∼2 nm resolutions and three-dimensions. Our study reveals a recurrent motif comprised of a thin (2–3 nm) filamentous core decorated with evenly spaced monomers or dimers of the αA-crystalline. By self-association this αA-crystalline motif constructs 3D-matrices that can adopt a myriad of geometric patterns. These matrices enfold the massive quantities of crystallines in fiber cells of the lens cortex and nucleus. It thus seems likely that to attain transparency the lens combines the long-range order of the cytoskeleton with the short-range order of dense soluble protein solutions.

## Methods

### Ethics Statement

Lenses of adult rats aged 12–14 weeks were used in strict accordance with regulations established by the Animal Care and Use Committee, known as the Chancellor's Animal Research Committee (ARC), at UCLA. The animals were anesthetized by halothane inhalation or Nembutal injection and sacrificed by decapitation (ARC # 1994-244-52).

### 1. Preparation of Specimens

Puncturing the capsules of lenses from three adult rats allowed harvesting fragmented fiber cells used in the experiments (∼200 µg total protein). The cell fragments were suspended in 50 µl of 10 mM HEPES pH 7.2 and centrifuged at low speed. The pellet was divided in four aliquots and suspended in: a) 200 µl of buffer, b) 190 µl of buffer plus 10 µl of 1 µg/µl solution of anti-αA-crystalline (9 mg/ml), c) 150 µl of buffer plus 50 µl of anti-αA-crystalline and d) 150 µl of buffer, 50 µl of antibody solution. They were incubated for 30 min at 4°C. A fourth aliquot (d) was suspended in 100 µl of secondary antibody conjugated to 5 nm diameter gold particles for 30 min at 4°C and washed by centrifugation. This fraction was then suspended in 100 µl of secondary antibody conjugated to 2 nm gold particles and incubated at 4°C for 30 min, washed in the same buffer. For thin sectioning electron microscopy, pellets were fixed in 3% glutaraldheyde in 0.1 M Na-Cacodylate buffer (pH 7.4) for 2 hours at room temperature. The post-fixation, dehydration, embedding, sectioning and staining steps were performed as described [22], [30]–[32].

### 2. Conical Tomography

We used the Gatan 650 Single Tilt Rotating Holder in a FEI Tecnai 12 Electron Microscope operated at 120 KV and a 2 k 2 k CCD Gatan camera. The thin sections were tilted first at a fixed angle (55°) and rotated by 5° steps through a complete 360° turn. Searching was done at 2,700 magnification and the regions of interest imaged by focusing ∼1.5 µm away from the region. Preliminary alignment of the series used the ImageJ software package [33]. A gold particle was selected as the centre of the conical series. The coordinates of 5–8 gold particles provided the orientation parameters (Euler angles and origin position) for computing a preliminary reconstruction using the weighted back-projection algorithm. This preliminary reconstruction was refined using strategies based on projection matching [34]–[36].

### 3. Gold Particle Maps

The large amplitude contrast and absence of “butterfly” artifacts allowed creation of 3D-maps comprised of only gold conjugates. To create these maps, we first calculated Z-projections with maximum intensity and measured the radii of the gold particles using ImageJ and the Analyze Particle utility. This type of analysis indicated that the radii extended through a continuum with not sharp boundaries between gold conjugates that should have measured 2 nm and 5 nm diameter. To circumvent this problem, we threshold the entire tomogram and used peak intensities to segment the gold particles using the watershed transform [37]. For each segmented particle, we computed the volume, the X,Y,Z coordinates of mass centers and the maximum intensity (0–255).

Based on the diameters (calculated from the volume) and peak intensities, the gold conjugates were classified into “yellow” and “blue” classes. Conjugates >4 nm diameter OR (Boolean operator) intensities >200 comprised the yellow class. Conjugates >2 nm in diameter OR intensities >150 comprised the blue class. Using the coordinates of the mass centers, each conjugate was compared to its near neighbor. A connection was assigned when the Euclidean distances measured less than 32 nm for yellow-yellow (red lines), 28 nm for yellow-blue (green lines) and 10 nm for blue-blue (brown lines). To fit Gaussian distributions to histograms of these three distances we used the program *fityk* [38].

### 4. Reference Space

To determine the structure of the assemblies containing the protein, small (128 128 50 pixels) volumes with a gold conjugate at its center were removed from the original reconstruction. Using the *Amira* software package, rotating the volume identified the tethers that connected the gold conjugate to neighboring protein assemblies. Once identified, the tethers were studied by: a) sectioning the volume into single-pixel slices along the X-Y, X-Z and Y-Z planes, b) calculating Z-projections, c) automatic and manual density segmentation, and d) calculating surface and volume rendering.

### 5. Analysis of Unlabeled Cells

The absence of gold conjugates prevents using Z-projections to identify the distribution of the αA-crystalline. Instead, we analyzed tomograms of unlabeled cells using the watershed transform by dividing the densities into particles of measurable volume. The approach was similar to the one used to study tomograms from cells labelled with antibody/gold conjugates after adjusting the thresholds for both volume and intensity. The adjustments were necessary because: a) the peak intensity of the particles was lower than that of gold conjugates and, b) the volume was variable, and c) large aggregates were not segmented into smaller particles by the transform. The particles were then classified as blue with diameters >2 nm but <3.5 nm and yellow with diameters >3.5 nm but <5 nm. Despite heterogeneity, it was possible to estimate an average volume of blue and yellow particles. The coordinates of the mass centres measured the Euclidean distances between blue and yellow particles and to characterize the molecular mass of the αA-crystalline particle.

Particles larger than 7 nm were lump together and referred to as “aggregates.” Two different strategies were used to exclude these “aggregates” from the analysis. One strategy involved a simple geometric constraint of aggregates occupying one corner of the extracted volume. The other strategy took advantage of the observation that the number of connections within aggregates was larger than among other groups of particles. We thus excluded particles linked to others with large number of connections.
